# Tracking the infection dynamics of *Fusarium oxysporum* in *Codonopsis pilosula* based on GFP labelling

**DOI:** 10.3389/fpls.2025.1586118

**Published:** 2025-08-15

**Authors:** Mingming Shi, Huixia Li, Wei Guo, Ning Luo, Jinghuan Chen, Yonggang Liu, Rui Liu, Zhenchuan Mao

**Affiliations:** ^1^ College of Plant Protection, Gansu Agricultural University/Biocontrol Engineering Laboratory of Crop Diseases and Pests of Gansu Province, Lanzhou, Gansu, China; ^2^ Institute of Plant Protection, Gansu Academy of Agricultural Sciences, Lanzhou, Gansu, China; ^3^ State Key Laboratory of Vegetable Biobreeding, Institute of Vegetables and Flowers, Chinese Academy of Agricultural Sciences, Beijing, China

**Keywords:** *Codonopsis pilosula*, *Fusarium oxysporum*, protoplast, GFP-labeled, infection dynamics

## Abstract

**Introduction:**

*Codonopsis pilosula* root rot, caused by *Fusarium oxysporum*, has caused severe damage to the *C. pilosula* industry. Due to the unclear pathogenic mechanisms of *F. oxysporum* on *C. pilosula*, the effective implementation of control measures has been greatly restricted.

**Methods:**

An efficient protoplast preparation and genetic transformation system was established for *F. oxysporum* FO-1, enabling real-time tracking of fungal colonization in *C. pilosula*. Single-factor experiments were conducted to determine optimal conditions, followed by response surface methodology to further optimize enzymatic parameters. PEG-mediated transformation was performed to generate GFP-tagged strains for infection tracking.

**Results:**

Single-factor experiments identified the optimal conditions as 12-hour-old mycelia treated with 0.7 M NaCl and 20 mg/mL driselase at 28°C and 180 rpm for 4 h. Response surface methodology optimized parameters to 188.24 rpm, 4.51 h, and 27.5°C, yielding 1.44 × 10^8^ CFU/mL protoplasts, representing a 30-fold improvement over single-factor optimization. PEG-mediated transformation produced 11 GFP-tagged strains, with FO-GFP-7 retaining wild-type morphology, growth rate, and pathogenicity. Microscopic observation revealed infection dynamics: conidia aggregated at the rhizome by 2 days post-inoculation (dpi), followed by phloem colonization at 4 dpi and vascular invasion at 6 dpi. Wound inoculation at the rhizome accelerated infection, consistent with field disease patterns linked to soil microfauna-induced injuries.

**Discussion:**

This study provides a robust platform for investigating F. oxysporum pathogenicity in *C. pilosula* and offers guidance on protective measures to maintain rhizome integrity during cultivation.

## 1 Introduction

*Codonopsis pilosula* (Franch.) Nannf., a member of the Campanulaceae family and the *Codonopsis* genus, is a traditional and valuable medicinal herb in China (Wang et al., 2023). In China, the planting area of *C. pilosula* is approximately 0.1 million hectares, with a total yield of 0.45 million tons (Yu, 2025). In traditional Chinese medicine, this plant is commonly employed to regulate the middle burner, enhance spleen function, and support lung health (Luan et al., 2021; Xin et al., 2012). Phytochemical investigations have revealed the presence of various bioactive compounds, including sterols, triterpenoids, glycosides, alkaloids, and polysaccharides (Wang et al., 1996). Contemporary pharmacological studies suggest that it possesses antioxidant, antimicrobial, and antitumor activities, along with the ability to modulate immune responses (Luo et al., 2007). In recent years, as research on the chemical components and action mechanisms of *C. pilosula* has deepened, its medicinal value been further developed and its economic value significantly increased. Consequently, *C. pilosula* presents broad development prospects (Sun et al., 2019; Xie et al., 2020). Root rot, caused by soil-borne pathogens, has been recognized as a critical limiting factor in *C. pilosula* cultivation, with documented yield reductions ranging from 10% to 60% (Lv et al., 2023). The symptoms are characterized by a rapid onset, with the above-ground stems and leaves turning yellow and the vascular bundles discoloring, leading to the decay of the main root and potentially resulting in the death of the entire field (Zhao et al., 2022). Fungi of the genus *Fusarium* are the primary pathogens, with *Fusarium oxysporum* being the predominant pathogen due to its higher field isolation frequency and pathogenicity compared to other pathogens (Zhao et al., 2021).

Currently, due to the unclear mechanisms by which *F. oxysporum* affects the roots of *C. pilosula*, there are few effective control measures in place. Therefore, it is urgent to clarify the key infection sites and dynamics of *F. oxysporum* for effective prevention and control (Wang et al., 2024). A crucial aspect of studying the pathogenic mechanisms of this pathogen involves establishing an efficient and stable genetic transformation system, which can be utilized to fluorescently label the pathogen and visualize its infection process (Amalamol et al., 2022). Consequently, the preparation of high-quality protoplasts becomes the most critical step in genetic transformation (Ning et al., 2022). The essential factor in preparing fungal protoplasts is the removal of the cell wall without compromising the integrity of the cell membrane (Wu et al., 2018). Researchers have found that enzymatic degradation of the cell wall yields intact protoplasts, with key influencing factors including mycelial age, types of stabilizing agents, the type of cell wall-degrading enzymes, and the conditions for enzymatic hydrolysis (Zhang et al., 2022). Different fungal species require distinct conditions for protoplast preparation (Wu and Chou, 2019), and variations may exist among different physiological races (Shin et al., 2019). Currently, there are limited reports on the preparation of protoplasts from *F. oxysporum* associated with root rot in *C. pilosula*, indicating a need for further exploration.

Fluorescent protein labeling has emerged as a critical tool for the real-time visualization of host-pathogen interactions, effectively addressing the limitations of conventional methods that hinder dynamic observation of infection processes *in planta* (Rajasekaran et al., 2008). The green fluorescent protein (GFP), which emits stable fluorescence under specific wavelengths (Lippincott-Schwartz and Patterson, 2003), facilitates non-invasive tracking of fungal colonization patterns. Since its first application in *Phytophthora parasitica* via protoplast transformation (Bottin et al., 1999), this technique has been extensively utilized to investigate filamentous fungal pathogens including *F. oxysporum*, *F. graminearum*, and *Alternaria alternata* (Crespo-Sempere et al., 2011; Zarzyńska-Nowak et al., 2024; Fu et al., 2024). Notably in *Fusarium* research, GFP-tagged strains have elucidated critical infection mechanisms. *F. oxysporum* f. sp. *cubense* preferentially invades banana root tips and lateral root wounds prior to systemic vascular colonization (Li et al., 2011). *F. proliferatum* demonstrates competitive root colonization in maize without precluding subsequent infections by *F. verticillioides* (Gaige et al., 2020). These findings demonstrate the technology’s capacity to resolve spatiotemporal infection dynamics, which is essential for developing targeted control strategies. The soil-borne pathogenic fungus *F. oxysporum* primarily causes disease by infecting the underground parts of plants; thus, research into its pathogenic mechanisms is of significant importance for crop protection. Current studies on the infection process of *Fusarium* species predominantly focus on crop systems such as tomatoes and cucumbers, where the fruit is the harvested product. In contrast, systematic research on economic crops like *C. pilosula*, where the rhizome is the harvested product, remains relatively scarce. Given that the infection sites of this pathogen significantly overlap with the main economic parts of *C. pilosula*, its pathogenic processes may possess unique mechanisms distinct from those of above-ground diseases. Therefore, elucidating the colonization dynamics and pathogenic patterns of *F. oxysporum* in the rhizome tissues of *C. pilosula* holds significant scientific value for enhancing the theoretical framework of soil-borne disease control in rhizome crops.

Currently, root rot disease caused by *F. oxysporum* is severe on *C. pilosula*, and controlling it in the field remains challenging (Zhao et al., 2021; Li et al., 2025). However, the effective application of control measures is significantly hindered by a lack of understanding regarding the infection sites and the infection process of this disease. In this study, we optimized the conditions for the genetic manipulation of *F. oxysporum* to obtain a green fluorescent-labelled strain, allowing us to trace the infection process and identify key invasion sites. The results provide valuable insights into the infection mechanisms of *F. oxysporum*, serving as a reference for field control strategies against root rot in *C. pilosula*.

## 2 Materials and methods

### 2.1 Strain and culture conditions

*F. oxysporum* FO-1 was collected, isolated, purified, and identified from the *C. pilosula* cultivation area in Tanchang County, Longnan City, Gansu Province, China. The strain FO-1 was cultured on potato dextrose agar (PDA) medium, which consists of 200.0 g of potato, 20.0 g of glucose, and 18.0 g of agar per liter of distilled water, followed by autoclaving at 121°C for 30 minutes, and incubated at 25°C.

For the collection of fresh mycelia, potato dextrose broth (PDB) medium was utilized, prepared by dissolving 24.0 g of PDB medium powder in 1000 mL of distilled water, autoclaved at 121°C for 30 minutes. Luria-Bertani medium (LB), composed of 10.0 g of peptone, 10.0 g of NaCl, and 5.0 g of yeast extract per 1000 mL of distilled water, was used to culture *Escherichia coli* Trelief 5α (Tsingke, Beijing, China) at 37°C for vector construction, supplemented with kanamycin at a concentration of 50 μg/mL. For protoplast regeneration, T-TOP medium was prepared by dissolving 0.5 g of KCl, 0.5 g of MgSO_4_·7H_2_O, 1.0 g of KH_2_PO_4_, 2.0 g of NaNO_3_, 200.0 g of sucrose, 20.0 g of glucose, and 1.0% agar powder in 1000 mL of distilled water, followed by autoclaving at 121°C for 20 min.

### 2.2 Preparation and optimization of conditions for FO-1 protoplasts

#### 2.2.1 Preparing of FO-1 protoplasts

Protoplasts were prepared from FO-1 using a modified protocol based on the methodology described by Vergara et al (Vergara et al., 2007). Fresh conidia were inoculated into PDB medium and incubated at 28°C. The harvested mycelia were sequentially treated with an osmotic stabilizer and digested using a wall-breaking enzyme. The resulting digestion mixture was filtered to remove undigested debris, followed by centrifugation at 2,000×g for 15 minutes at 4°C to collect the protoplasts. The purified protoplasts were resuspended in STC buffer (1.2 M sorbitol, 50 mM CaCl_2_, 10 mM Tris-HCl, pH 7.5) and stored at 4°C for subsequent transformations.

#### 2.2.2 Effects of different experimental factors on protoplast yield

The experiment was conducted to compare and screen the mycelial age, types of stabilizing agents, types of cell wall-degrading enzymes, and enzymatic hydrolysis conditions (including incubation time, incubation temperature, and rotations per minute) during the preparation of FO-1 protoplasts, with the aim of optimizing the conditions for the preparation.

Protoplast yield optimization required precise control of the mycelial physiological states, as suboptimal mycelial age can lead to reduced protoplast yield due to either cell wall thickening or structural fragility (Zhou et al., 2011). Mycelia cultured for 9 to 13 hours (h) in PDB medium were digested using 20 mg/mL driselase in a 0.7 M NaCl solution at 28°C with a rotation speed of 180 rpm for 3 h. Protoplast quantification, performed via hemocytometer analysis, revealed that 11-hour cultures were optimal for maximal viable protoplast production.

The stabilizing agent is a crucial factor influencing the preparation of protoplasts, with its concentration significantly affecting protoplast yield (Choi et al., 2024). Mycelia aged 11 hours were treated with osmotic stabilizing agents at various concentrations of 0.6 mol/L (A) and 0.8 mol/L (B) glucose, 0.6 mol/L (C) and 0.8 mol/L (D) mannitol, 0.6 mol/L (E) and 0.7 mol/L (F) NaCl, and 0.6 mol/L (G) and 1.2 mol/L (H) KCl. Enzymatic digestion was performed for 3 h at 28°C with a shaking speed of 180 rpm. The effects of eight different stabilizers on protoplast production were analyzed to identify the most effective stabilizer.

Different wall-breaking enzymes exhibit varying enzymatic hydrolysis effects on mycelium (Kuang et al., 2002). Four wall-breaking enzymes were selected for this experiment: driselase (A), cellulase (B), snail enzyme (C) and lysozyme (D), each at a concentration of 20 mg/mL, in eight combinations (A, B, C, D, B+C, B+D, C+D, B+C+D). The optimal wall-breaking enzymes were determined based on protoplast yield.

The optimal enzymatic lysis conditions during protoplast preparation vary significantly among different strains (Islas-Lugo et al., 2016). To obtain high-quality protoplasts through enzymatic degradation, it is essential to optimize the specific conditions for each particular strain. Fresh mycelia were collected from 11 hours of shake cultivation using 0.7 mol/L NaCl as a stabilizing agent, followed by the addition of driselase. Various incubation times (2, 3, 4, 5, and 6 h), rotations per minute (120, 140, 160, 180, and 200 rpm/min), and incubation temperatures (20, 24, 28, 32 and 36°C) were tested to determine the optimal enzyme digestion conditions for protoplast preparation.

#### 2.2.3 Optimization of conditions for *F. oxysporum* FO-1 protoplasts using response surface methodology

Based on the results of the single-factor experiment, three factors and their respective ranges of influence were selected (**Supplementary Table S1**). Subsequently, a Box-Behnken test was designed utilizing Design-Expert 13 software, resulting in a total of 17 experimental conditions (refer to **Supplementary Table S2**). Each condition was tested in triplicate to ensure the reliability of the results.

### 2.3 Establishment of a genetic system for *F. oxysporum* FO-1 GFP marker

#### 2.3.1 Antibiotic resistance screening

The wild-type strain FO-1 was inoculated onto PDA medium and cultivated for five days at 25°C. Subsequently, it was transferred to PDA plates containing varying concentrations of hygromycin B: 10, 20, 40, 80, and 160 μg/mL, with a control plate that did not contain hygromycin B. This experiment was repeated three times. Colony growth was assessed after five days of culture at 25°C, to determine the lowest inhibitory concentration (Su et al., 2021).

#### 2.3.2 PEG-mediated transformation

Protoplast transformation was conducted using an optimized polyethylene glycol (PEG)-mediated protocol adapted from Luo et al. (Luo et al., 2023). The transformation mixture comprised 5 μg of the pch-SGFP plasmid, which carries the green fluorescent protein (GFP) as a reporter gene and hygromycin B resistance (Hyg) as a selectable marker; this plasmid was kindly provided by Prof. Zhenchuan Mao; from the Institute of Vegetables and Flowers, Chinese Academy of Agricultural Sciences. Additionally, 10 μL of aurintricarboxylic acid (100 mM) was included in the TEC buffer (10 mM Tris-HCl, 30 mM CaCl_2_, 25 mM EDTA, pH 7.5) and incubated with freshly prepared protoplasts (1×10^7^ cells/mL) on ice for 20 min. Following centrifugation at 12,000×g for 2 min, the pellet was sequentially treated with 160 μL of 60% PEG 4000 and 1 mL of STC buffer, with 20 min incubations at 25°C after each addition. Transformed protoplasts were then resuspended in 200 μL of STC buffer and plated on T-TOP regeneration medium. After 12 to 20 h of incubation at 25°C, transformants were selected by over laying with PDA medium containing 100 μg/mL of hygromycin B. Single colonies were isolated and transferred to PDA screening medium with hygromycin B for incubation at 25°C. The single colonies that continue to grow were regarded as candidate transformants.

### 2.4 Identification of transformant

#### 2.4.1 Phenotypic analysis and genetic stability testing of transformant

The candidate transformants were inoculated onto hygromycin B screening plates and cultured for 4 days at 25°C, followed by six successive cultures. Subsequently, the marginal mycelium of the colony was transferred to a standard PDA plate for five additional cultures, and then incubated at 25°C for 6 days. The phenotype of the colony was observed, and the growth rate and spore production were measured. The wild-type strain served as the control; if the transformants exhibited normal growth, it indicated their genetic stability.

#### 2.4.2 Fluorescence detection of transposons

The screened transformants were transferred to PDA medium and cultured for five days. Mycelia from the transformants were then picked onto a slide and observed for green fluorescence using a fluorescence microscope (Carl Zeiss Axio Scope 5, Germany).

#### 2.4.3 DNA identification of transformant

The transformants were selected and cultured on PDA for genomic DNA extraction. GFP-specific primers GFP-F/R (5′-CCATACTCCATCCTTCCCATCCC-3′ and 5′-CCATACTCCATCCTTCCCATCCC-3′) were employed for PCR amplification, and the resulting PCR products were analyzed using a 1% agarose gel (Sun et al., 2024).

#### 2.4.4 Pathogenicity assay

The roots of *C. pilosula* were surface-sterilized using 75% ethanol, followed by triple rinsing in sterile water and UV treatment for 30 minutes in a clean bench environment (Zhao et al., 2024). Mycelial plugs from the transformant FO-GFP or the wild-type FO-1 were aseptically placed on the root surfaces, ensuring that hyphal mats were in contact with the epidermal tissues. The inoculated roots were maintained at 25°C with 85% relative humidity. Disease progression was assessed at 6 dpi through a comparative analysis of lesion expansion rates and the severity of vascular discoloration between the transgenic and wild-type strains.

### 2.5 Studies on the process of infection of *C. pilosula* by fluorescent transformants

#### 2.5.1 Inoculation experiment

FO-GFP were cultured on PDA plates for 7 days at 25°C, and conidial suspensions were standardized to 1×10^7^ CFU/mL using hemocytometric quantification. Two-year-old *C. pilosula* cv. Wei Dang No. 1 roots were sterilized using 75% ethanol and UV exposure. For tissue-specific infection analysis, sterilized roots were subjected to five inoculation modalities via wound/immersion (Koima et al., 2023): 1) rhizome puncture, 2) root tip immersion, 3) proximal root immersion, 4) mid-root immersion, and 5) whole-root immersion (**Supplementary Figure S1**). Inoculated roots were incubated at 25°C/85% with 85% relative humidity (RH). The dynamics of GFP fluorescence were captured using a fluorescence microscope in accordance to with hypha colonization rates (**Supplementary Figure S2**).

#### 2.5.2 Histopathological analysis

Root segments from inoculation sites were aseptically collected at 2, 4, and 6 dpi for spatial-temporal infection analysis. The samples were fixed in 4% paraformaldehyde (w/v in PBS, pH 7.4) for 24 h at 4°C, followed by triple rinsing with sterile phosphate-buffered saline (PBS) to eliminate surface-adherent conidia. Transverse and longitudinal sections of *C. pilosula* were prepared using a disposable scalpel and subsequently mounted on glass slides. The GFP-tagged hyphal colonization patterns were observed under a fluorescence microscope.

#### 2.5.3 Glasshouse pot experiment

Based on the results obtained, *C. pilosula* cv. Wei Dang No. 1 seedlings were transplanted into pots filled with sterile soil and subjected to targeted rhizome inoculation. Insect needles were employed to puncture the rhizome, and a spore suspension of *F. oxysporum* at a concentration of 1×10^7^ CFU/mL was injected near the rhizome head of the *C. pilosula*. The seedlings were moisturized for three days and then incubated at 25°C for 45 days. After the incubation period, the seedlings were removed for observation.

### 2.6 Data analysis

Data processing was conducted using Microsoft Excel 2016, and all analyses were performed with SPSS 24.0 (IBM SPSS Statistics 24.0, USA). Duncan’s new complex polar method was employed to assess the significance of differences (p < 0.05). Additionally, response surface multiple regression analysis was carried out using Design-Expert 13 software.

## 3 Results

### 3.1 Optimal conditions for *F. oxysporum* FO-1 protoplasts

#### 3.1.1 Effects of different experimental factors on protoplast yield

**Figure 1:**
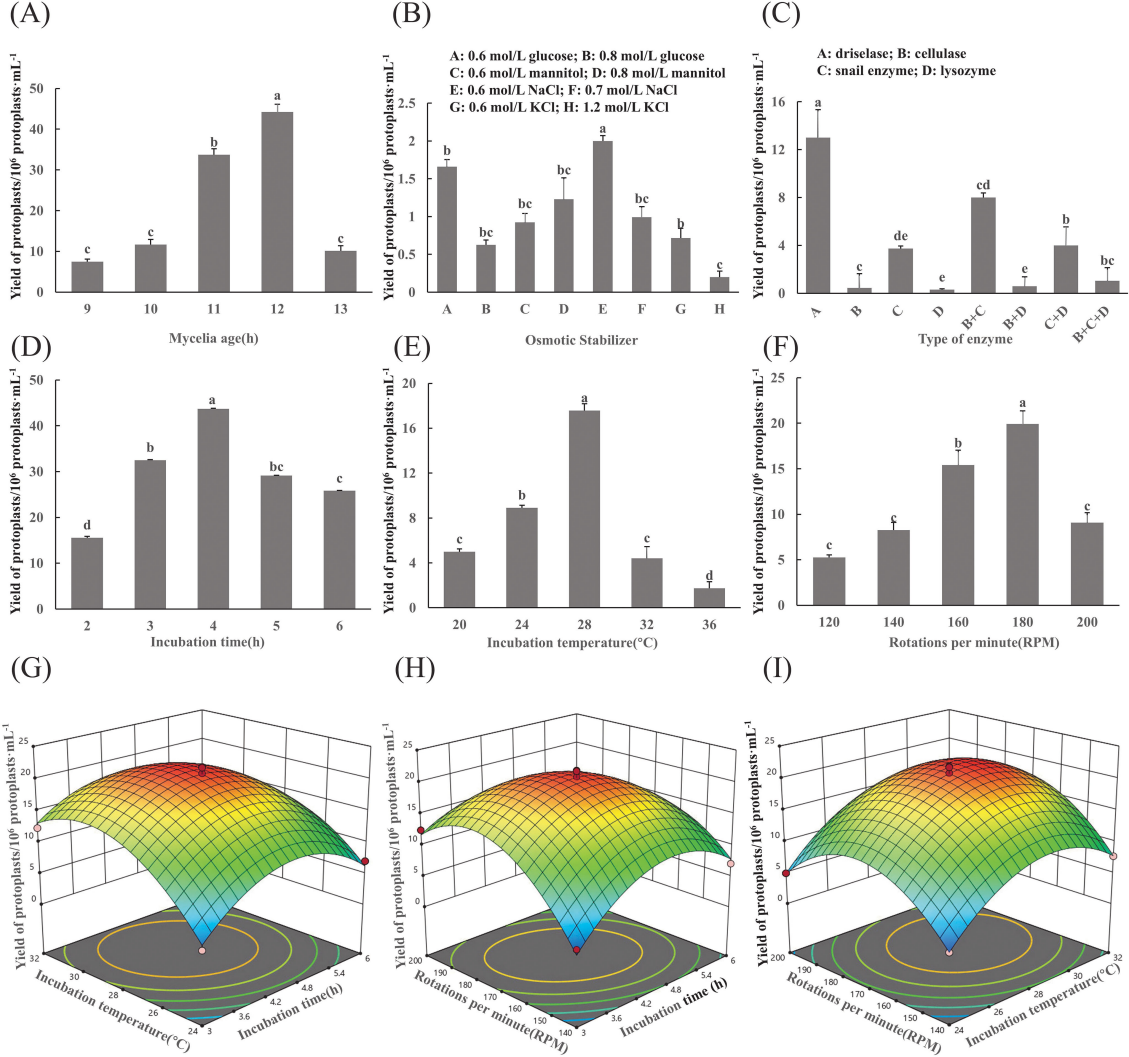
Optimization of protoplast preparation conditions for *F. oxysporum*. **(A-F)** Effects of different experimental factors on protoplast yield of *F. oxysporum* FO-1. **(G-I)** Response surface methodology-driven optimization of *F. oxysporum* FO-1 protoplast preparation conditions. **(A)** Mycelial age, **(B)** Osmotic stabilizers, **(C)** Type of enzyme, **(D)** Incubation time, **(E)** Incubation temperature, **(F)** Rotations per minute, **(G)** Incubation temperature and incubation time, **(H)** Incubation speed and incubation time, **(I)** Incubation speed and incubation temperature. Different letters indicate significant differences according to one-way ANOVA followed by Tukey's multiple comparisons test (P < 0.05).

The results of the mycelial age indicated that a greater number of mycelia were obtained after 12 h of incubation, and the final yield of protoplasts was significantly higher than that of the other treatments (**Figure 1A**). When 0.7 mol/L NaCl was used as an osmotic stabilizer, the highest protoplast yield of 2×10^7^ protoplasts/g was achieved, which was significantly greater than that of the other treatments (**Figure 1B**).

Cell wall-degrading enzymes are essential for protoplast preparation. In this experiment, driselase demonstrated the highest enzymatic efficiency, yielding 1.3×10^7^ protoplasts/g, which was significantly higher than that of cellulase, glusulase, and lysozyme (*p < 0.05*). Lysozyme exhibited the lowest efficiency, producing only 3.0×10^5^ protoplasts/g (**Figure 1C**).

The results revealed that different enzymatic digestion times and temperatures had effects on yield (**Figures 1D, E**). T The protoplast yield peaked at 4.37×10^7^/g after enzymatic digestion 4h and at 28°C, which was significantly higher than that of the other treatments (*p*<0.05). When the rotational speed exceeded 180 rpm, protoplast yields dramatically declined (**Figure 1F**).

#### 3.1.2 Condition optimization results of Box-Behnken test

Based on preliminary single-factor experiments, a Box-Behnken design was implemented with incubation time (A), temperature (B), and rotations per minute (C) as independent variables, while protoplast yield (1×10^8^ CFU/mL) served as the response variable. The effects of the interactions among these factors on the response value were also investigated. The experimental results are presented in **Table 1**. Second-order polynomial regression analysis (Design Expert 12.0) generated the predictive model:

**Table 1:** The results of Box-Behnken design test.

Test no.	A: Incubation time (h)	B: Incubation temperature (°C)	C: Rotations per minute (rpm/min)	D: Protoplast yield (1×10^7^ CFU/mL)
1	-1	-1	0	3
2	-1	0	1	12.5
3	0	-1	1	4.975
4	0	1	-1	7.75
5	0	0	0	20.875
6	0	0	0	19.875
7	1	-1	0	7.05
8	0	0	0	21.875
9	0	1	1	12
10	1	0	1	4.375
11	0	0	0	19.5
12	0	-1	-1	2.6
13	-1	0	-1	3.475
14	1	0	-1	5.1
15	-1	1	0	12.342
16	1	1	0	10.025
17	0	0	0	21.75


$$\begin{array}{c} R=20.77-0.3459A+3.06B+1.62C-1.59AB\\ -2.94AC-0.4688BC-6.91A^{2}-6.35B^{2}-7.59C^{2}. \end{array}$$

Statistical validation through ANOVA revealed model significance (F-value=64.88, p<0.0001) with excellent predictability (adjusted R²=0.9882), explaining 98.82% of the response variation. The nonsignificant lack of fit (p=0.3628) confirmed model adequacy (**Table 2**). Main effects analysis identified temperature (B, p<0.01) and quadratic terms (A²/B²/C², p<0.01) as critical determinants of protoplast yield.

**Table 2:** Variance analysis of regression model.

Source	Sum of squares	df	Mean square	F-value	P-value	Significance
Model	790.66	9	87.85	64.88	0.0001	**
A	0.9570	1	0.9570	0.7068	0.4283	–
B	74.98	1	74.98	55.38	0.0001	**
C	20.88	1	20.88	15.42	0.0057	**
AB	10.13	1	10.13	7.48	0.0291	*
AC	34.52	1	34.52	25.49	0.0015	**
BC	0.8789	1	0.8789	0.6491	0.4469	–
A^2^	168.17	1	168.17	124.2	0.0002	**
B^2^	169.83	1	169.83	125.43	0.0010	**
C^2^	242.74	1	242.74	179.27	0.0001	**
Residual	9.48	7	4.11	–	–	–
Lack of fit	4.87	3	1.62	1.41	0.3628	–
Pure error	4.61	4	1.15	–	–	–
Cor total	931.48	16	–	–	–	–
Model	790.66	9	87.85	64.88	0.0001	**

The difference was not significant (P>0.05); *the difference was significant (*P*<0.05); **the difference was very significant (P<0.01); - was no data.

Three-dimensional response surface analysis demonstrated interaction hierarchies: AC > AB > BC, evidenced by the gradient steepness in contour plots (**Figures 1G–I**). Model optimization predicted a maximal yield (1.84×10^8^ CFU/mL) at 188.24 rpm, 4.51 h, and 27.50°C. Practical validation under adjusted conditions (190 rpm, 4.5 h, 28°C) achieved 1.44×10^8^ CFU/mL, demonstrating 78.3% congruence with theoretical predictions. The ≤15% deviation between empirical and predicted values confirmed model robustness for industrial-scale protoplast production.

In summary, the optimal conditions for the preparation of *F. oxysporum* FO-1 protoplasts, as determined by single-factor experiments, were as follows: 0.7 mol/L NaCl as an osmotic stabilizer, hyphae pre-cultured for 12 h, enzymatic digestion with 20 mg/mL lysing enzymes at 28°C and 180 rpm for 4 h. Under these conditions, a high yield of viable protoplasts was obtained.

### 3.2 Screening of resistance concentration of FO-1 to hygromycin B

**Figure 2:**
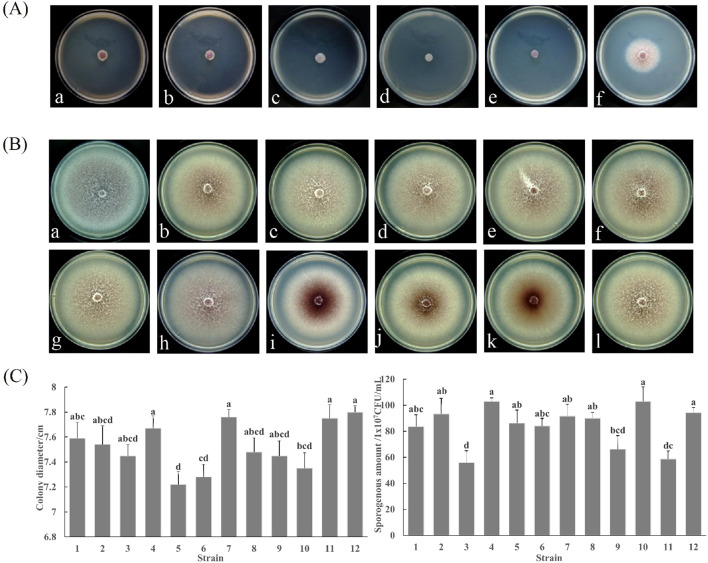
Phenotypic characterization of *F. oxysporum* FO-1 and its GFP transformants. **(A)** Response of *F. oxysporum* to hygromycin B at different concentrations, (a) 10 μg/mL; (b) 20 μg/mL; (c) 40 μg/mL; (d) 80 μg/mL; (e) 160 μg/mL; (f) 0 μg/mL. **(B)** Colony morphology between wild type strain FO-1 and transformant FO-GFP-1~11, (a) Wild type strain FO-1; (b-l) Transformant FO-GFP-1~11. **(C)** Colony growth rate and sporogenous amount of wild type FO-1 and transformant FO-GFP. 1–11 is the transformant FO-GFP-1~11; 12 is a wild strain FO-1. Different letters indicate significant differences according to one-way ANOVA followed by Tukey's multiple comparisons test (P < 0.05).

The results of FO-1 in response to hygromycin B are illustrated in **Figure 2A**. The growth of FO-1 exhibited a gradual inhibition correlating with the increasing concentration of the antibiotic. At a concentration of 80 μg/mL, the growth of strain FO-1 was markedly reduced (**Figures 2A**, d), while complete inhibition was observed at 160 μg/mL of hygromycin B (**Figures 2A**, e). Consequently, a concentration of 160 μg/mL was selected as the screening threshold.

### 3.3 Acquisition of FO-GFP transformants

#### 3.3.1 Phenotypic characterization of GFP-Tagged transformants

Eleven *F. oxysporum* transformants (FO-GFP-1 to FO-GFP-11)) stably expressing GFP were evaluated against the wild-type strain FO-1 across three phenotypic parameters to identify optimal candidates for pathogenesis studies.

Colony Morphology: After a 7-day culture on PDA, FO-GFP-7 exhibited pigmentation and hyphal density nearly identical to those of the wild-type FO-1 (**Figure 2B**, a vs. **2B**, h). Transformants FO-GFP-8 to FO-GFP-10 displayed centralized myelinization accompanied by peripheral hyphal thinning (**Figure 2B**, i-k), while FO-GFP-1 to FO-GFP-6 and FO-GFP-11 showed reduced pigmentation (**Figure 2B**, b-g, l).

Mycelial Growth Rate: All transformants demonstrated slightly reduced growth rates compared to the wild-type (7.80 cm diameter). FO-GFP-4 (7.67 ± 0.12 cm), FO-GFP-7 (7.76 ± 0.15 cm), and FO-GFP-11 (7.75 ± 0.18 cm) exhibited nonsignificant differences (p > 0.05) from wild-type FO-1 (**Figure 2C**).

Conidiation Capacity: The Wild-type FO-1 produced 9.43×10^8^ CFU/mL. FO-GFP-2 (9.34×10^8^) and FO-GFP-7 (9.17×10^8^) matched the productivity of the parental strain, whereas FO-GFP-4 (1.03×10^9^) and FO-GFP-10 (1.01×10^9^) exhibited hyper-conidiation (**Figure 2C**).

Integrated analysis identified FO-GFP-7 as the optimal transgenic line, as it preserved the wild-type morphology, growth kinetics, and sporulation capacity.

#### 3.3.2 Fluorescence detection of transformants

**Figure 3:**
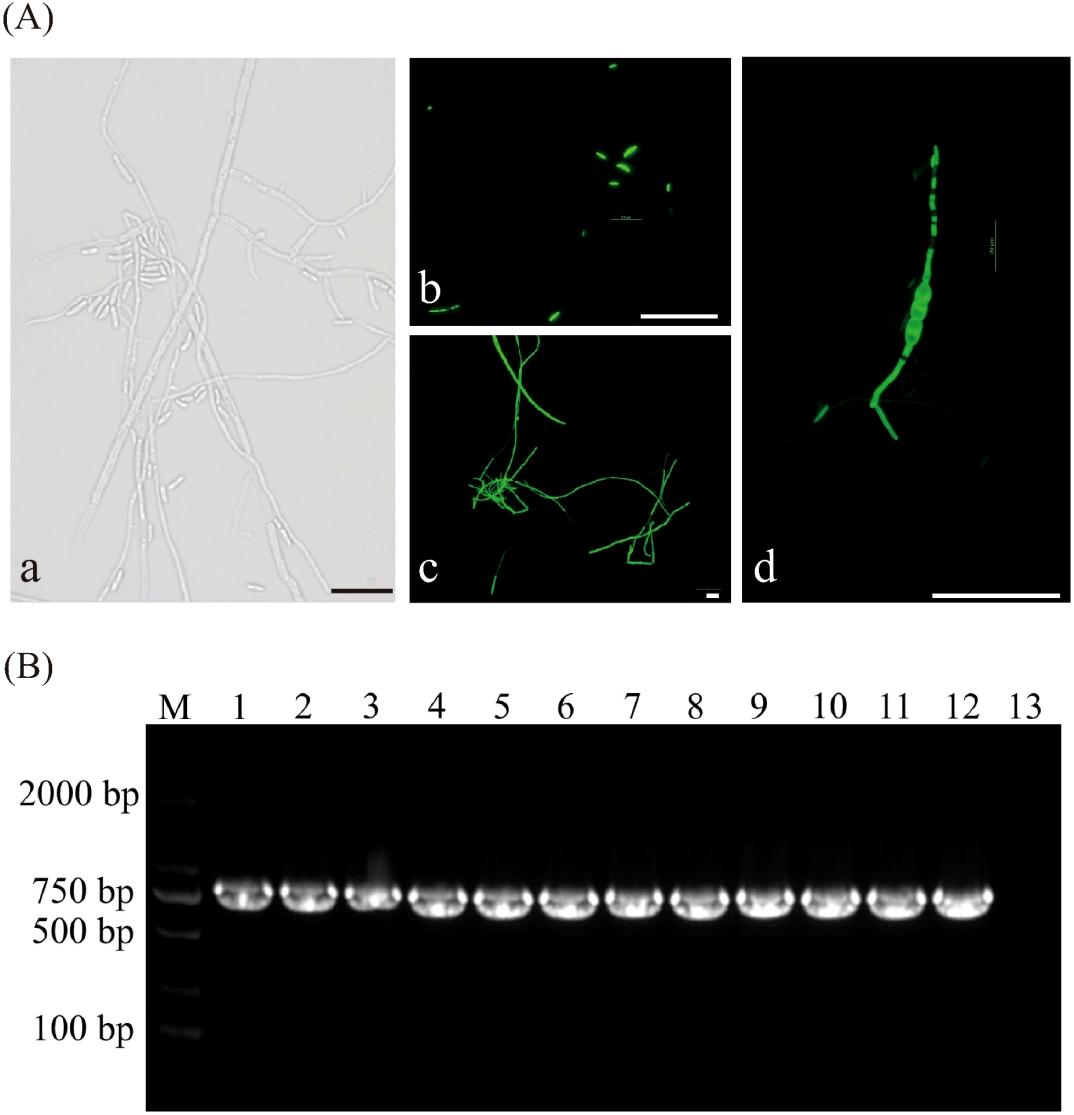
Assessment of the genetic stability of GFP transformants. **(A)** Fluorescent detection in transformant strains, (a) Bright field images of transformant strain, (b-d) Distribution of GFP green fluorescence in spores and hyphae of transformant strains at different developmental stages, (b) Conidiospores, (c) Mycelium, (d) Chlamydospore. **(B)** Agarose gel electrophoresis of PCR products for screening of GFP gene in transformants of *F. oxysporum*, M: DL 2000 Maker; Lanes 1 to 11: Transform; Lane12: Positive control (plasmid); Lane13: Negative control. All scale bars represent 50 µm.

To verify the *in vivo* fluorescence expression of the transformants, hyphae samples were collected, prepared as temporary slides, and observed under a fluorescence microscope. The results indicated that the mycelia and spores of the transformants emitted stable green fluorescence, whereas no fluorescence was detected in the bright field (**Figure 3A**).

#### 3.3.3 PCR detection

After sub-cultivation, all eleven transformants of the FO-1 exhibited normal growth on PDA plates containing hygromycin B, indicating that the pch-SGFP plasmid DNA had been successfully integrated into the genomes of the strains. A 643 bp fragment (lanes 1 to 11) from the *F. oxysporum* FO-1 strain was amplified using GFP-F/R (**Figure 3B**), demonstrating that the GFP plasmid was effectively transferred into the wild-type FO-1 strain with good stability.

#### 3.3.4 Pathogenicity test of transformants

**Figure 4:**
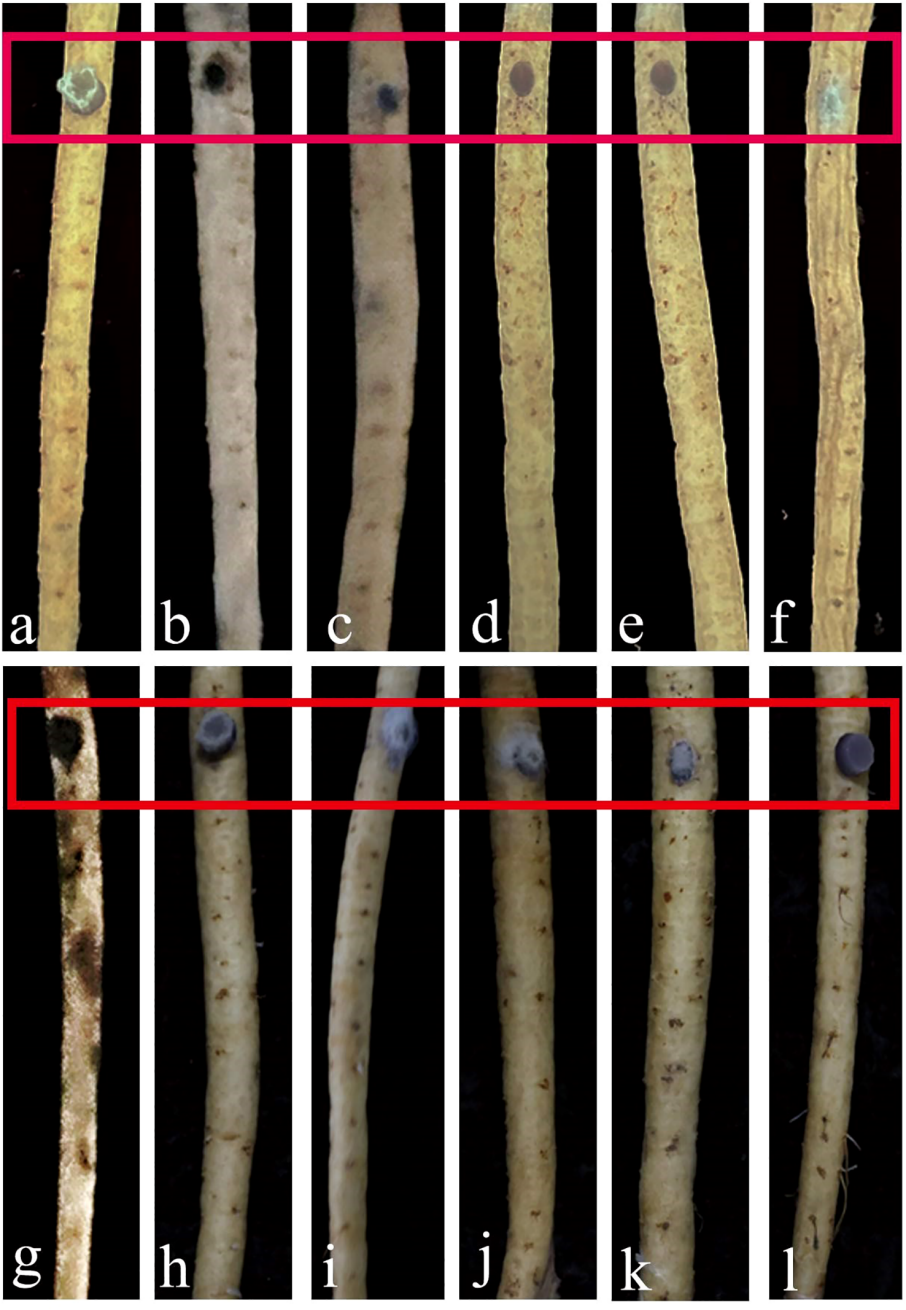
Comparison of pathogenicity between wild-type FO-1 and transformant FO-GFP-1~11. **(a)** Pathogenicity of wild type strain FO-1; **(b-l)** Pathogenicity of transformant FO-GFP-1~11. Red boxes indicate the areas of infection.

The pathogenicity results of *F. oxysporum* transformants on *C. pilosula* revealed three distinct virulence phenotypes at 6 dpi (**Figure 4**). The pathogenicity of FO-GFP-7 was demonstrated to be equivalent to that of the wild-type strain, exhibiting symptoms of root rot (**Figures 4h** vs. **4a**). Hypo-virulent strains, including FO-GFP-2 to -4 and FO-GFP-11, displayed attenuated pathogenesis, characterized by superficial desiccation without vascular penetration. In contrast, hyper-virulent strains FO-GFP-1, -5, -6, and -8 to -10 resulted in accelerated tissue maceration, with FO-GFP-6 exhibiting extreme virulence accompanied by necrotic myelinization (**Figure 4g**). Despite the enhanced pathogenicity of FO-GFP-6, FO-GFP-7 was prioritized for subsequent studies due to its preserved wild-type virulence dynamics and stable GFP expression.

Among the eleven transformants, FO-GFP-7 demonstrated the highest similarity to the wild-type FO-1 in terms of morphology, growth, sporulation, and virulence, and was therefore selected for further tests.

### 3.4 Infection process of *F. oxysporum* on *C. pilosula* based on GFP marker

#### 3.4.1 Infection process

**Figure 5:**
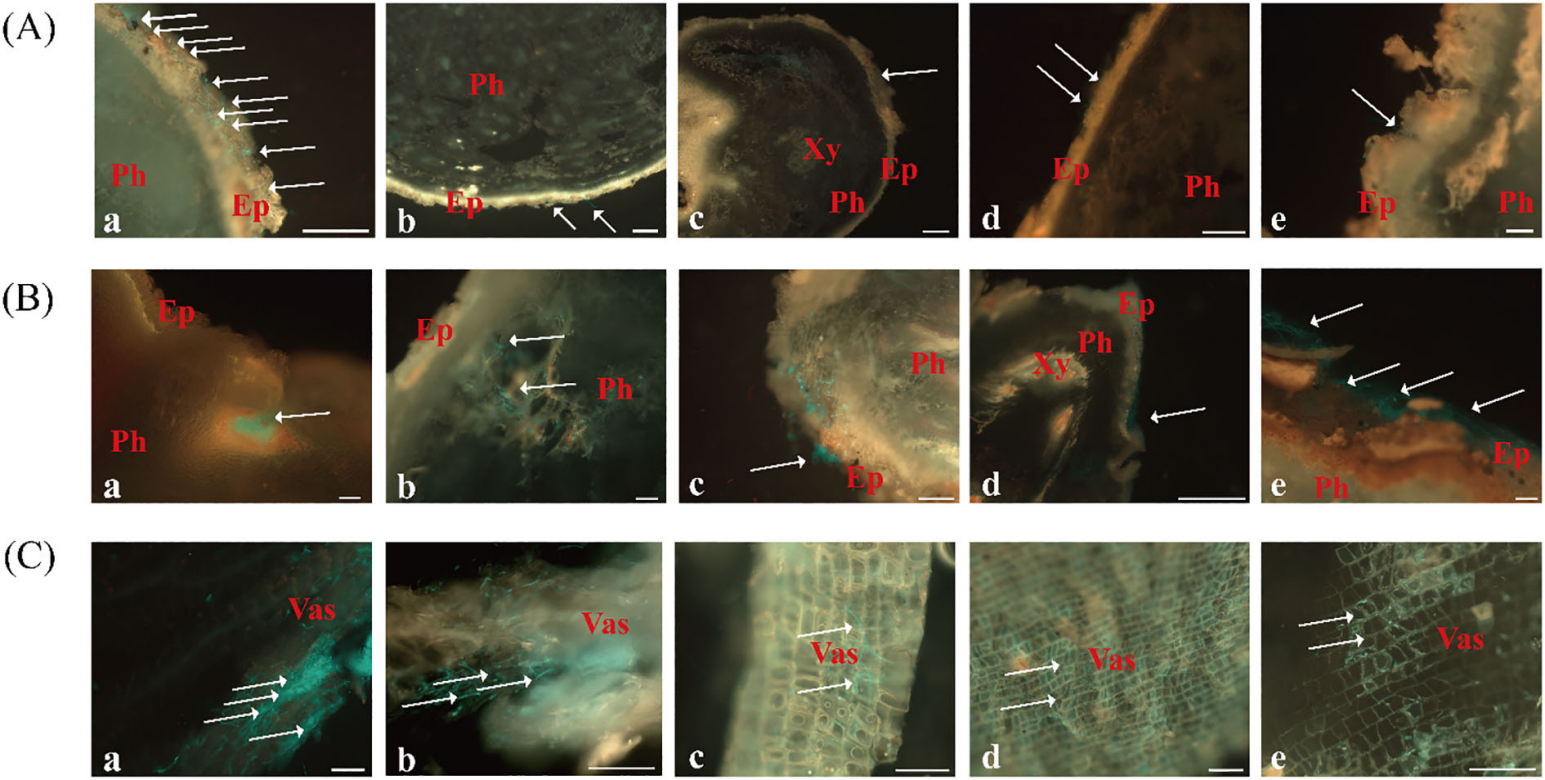
Colonization and expansion dynamics of FO-GFP strain in *C. pilosula* at 2, 4, and 6 dpi under different inoculation methods. **(A)** 2 dpi, Scale bars = 200 μm; **(B)** 4 dpi, Scale bars = 100 μm; **(C)** 6 dpi, Scale bars = 100 μm. Inoculation methods: (a) Rhizome puncture; (b) Root tip puncture; (c) Proximal root puncture; (d) Mid-root puncture; (e) Whole-root immersion. Ep, epidermis; Ph, phloem; Xy, xylem; Vas, vascular bundle.

The infection dynamics of *F. oxysporum* on *C. pilosula* exhibited significant variations depending on the inoculation method and site. At 2 dpi, aggregated spores were primarily observed on the root epidermis and numerous spores germinated into hyphae and accumulated within the cork layer in rhizome puncture inoculation (**Figures 5A**-a). In contrast, inoculations at root tip, proximal root, and mid-root puncture led to fewer spores on the epidermis, and only a small number spores germinated into hyphae, resulting in delayed infection compared to rhizome inoculation (**Figures 5A**, b-d). In the whole-root immersion treatment, spores were barely visible on the root surface (**Figures 5A**-e).

By 4 dpi, spore germination and hyphal penetration through the cork layer became evident in most treatments (**Figure 5B**). In rhizome-punctured plants, extensive hyphal proliferation was observed within the phloem, with dense fungal clusters detected in longitudinal sections (**Figures 5B**-a). Other inoculation methods showed only limited hyphal expansion, with sparse hyphae reaching the phloem region. Notably, symptoms of tissue softening and decay were exclusively observed in rhizome-inoculated specimens, whereas plants under other treatments retained intact surface tissues without visible deterioration (**Figures 5B**, b-e).

At 6 dpi, rhizome-inoculated plants exhibited severe disease progression, characterized with colonization at the vascular bundles, tissue browning, and advanced rot (**Figures 5C**-a). Plants inoculated through puncture at the root tip, proximal root, and mid-root sites showed moderate phloem colonization, minimal vascular bundle invasion, and absence of tissue maceration (**Figures 5C**, b-d). In contrast, plants inoculated by immersion showed the slowest infection rate and the least phloem colonization (**Figures 5C**-e).

#### 3.4.2 Pathogenicity validation of *F. oxysporum* via rhizome inoculation in *C. pilosula*

**Figure 6:**
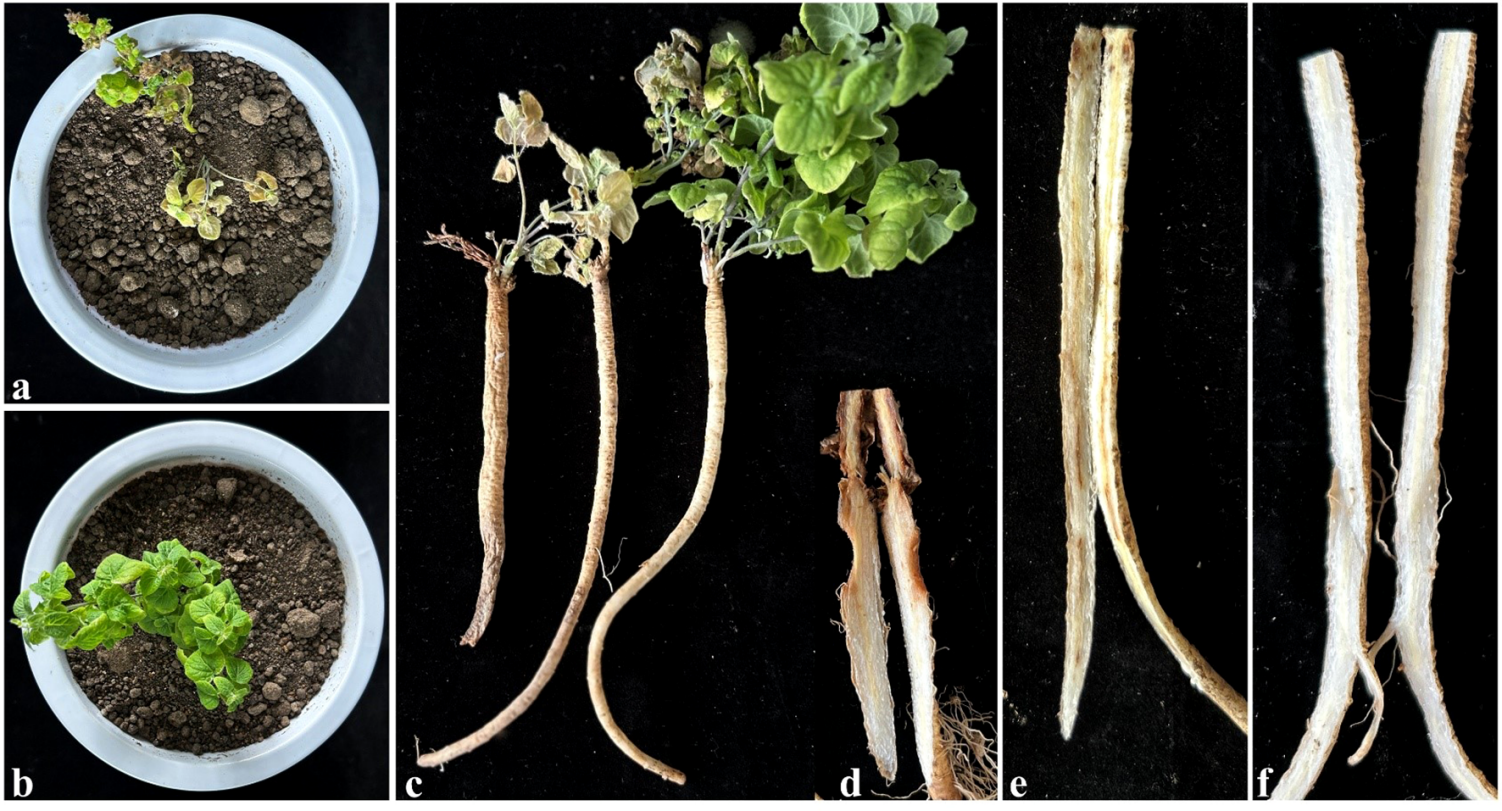
Potted experiment to verify the infection sites. **(a)** The shoot of diseased plants; **(b)** The shoot of control plants, **(c)** Control and diseased *C. pilosula* whole plant images; **(d, e)** Symptoms of the underground parts of diseased *C. pilosula*, **(f)** The underground parts of control.

After inoculated 42 days, plants exhibited severe aboveground symptoms, including leaf yellowing and desiccation (**Figures 6a**), which sharply contrasted with the healthy foliage of the controls (**Figure 6b**). The inoculated roots displayed softening around the rhizomes, accompanied by epidermal detachment (**Figure 6c**). Longitudinal sections revealed extensive browning of the vascular bundle, partial root rot, and loss of primary root integrity, mirroring the disease phenotypes observed in the field (**Figures 6d, e**). In contrast, control plants maintained intact root systems with white, non-discolored vascular tissues (**Figure 6f**). These results conclusively demonstrate that inoculation with rhizome puncture induces systemic infection in *C. pilosula*, recapitulating natural disease progression under controlled conditions.

The rhizomes were identified as the most susceptible site for *F. oxysporum* infection, exhibiting faster pathogen invasion and more severe symptoms compared to other parts of roots.

## 4 Discussion

### 4.1 Efficient preparation and genetic transformation of *F. oxysporum* protoplasts: a multivariate optimization approach

The efficient preparation of fungal protoplasts is critical for advancing genetic manipulation in filamentous fungi (Mózsik et al., 2019). In this study, we found that mycelial age, types of stabilizing agents, the type of cell wall-degrading enzymes, and enzymatic hydrolysis conditions significantly affected protoplast yield. Optimal conditions were identified as follows: 12-h-old logarithmic-phase mycelia treated with 20 mg/mL driselase in a 0.7 M NaCl solution, followed by 4 h incubation at 28°C with 180 rpm agitation. This result generally agrees with studies on other strains of the genus *Fusarium* (Mi et al., 2013). Notably, Xiao et al. (2009) demonstrated that protoplast preparation for *F. oxysporum* f. sp. *niveum* strain FOV-135 required a combination of lysing enzyme and cellulase, suggesting potential strain-specific variations in cell wall susceptibility. This underscores the necessity for tailored optimization for different *Fusarium* strains.

To enhance preparation efficiency, a Box-Behnken design-based response surface methodology (RSM) was employed for multivariate optimization. Mathematical modeling predicted optimal enzymatic conditions of 188.24 rpm, 4.51 h, and 27.50°C, resulting in a protoplast yield of 1.44×10^8^ CFU/mL, which represents a 30-fold enhancement over single-factor optimization. This underscores the superiority of multivariate strategies in bioprocess optimization.

Using the optimized protocol, we observed that the FO-GFP-7 transformant retained wild-type characteristics, indicating stable genomic integration of the GFP cassette without disrupting essential metabolic pathways. This finding is consistent with reports on *F. oxysporum* f. sp. *cubense* (Wu et al., 2016) and tomato wilt pathogens (Aragona et al., 2021).

**Figure 7:**
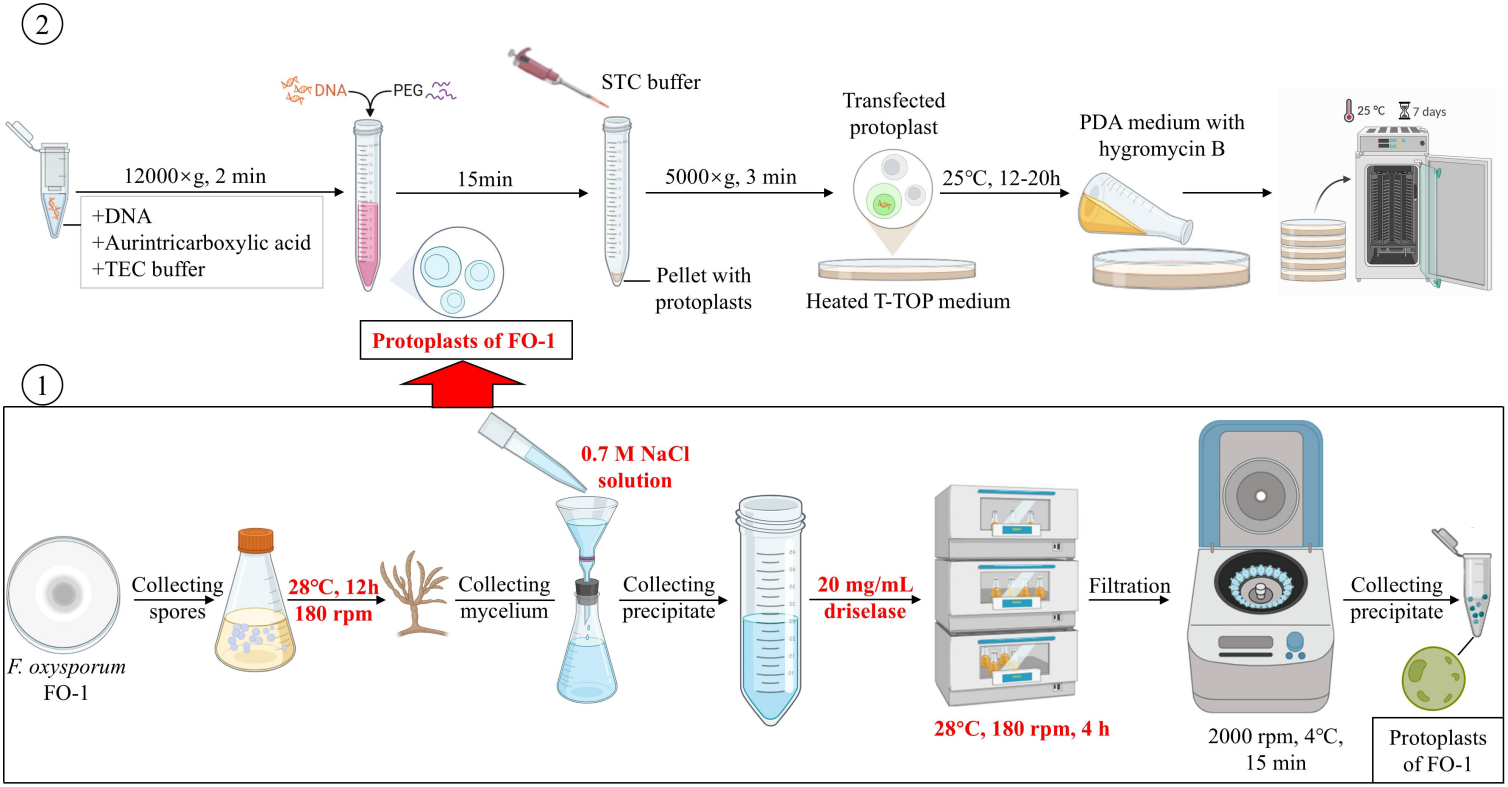
The standardized procedure for transformation of *F. oxysporum* FO-1 by using PEG-calcium-competent protoplasts.

This system was demonstrated stable fluorescence signals and enabled real-time visualization of *F. oxysporum* colonization in *C. pilosula*. This approach mirrors methodologies applied to *Phomopsis asparagi* (Zhang et al., 2018), validating its applicability for *in vivo* studies of pathogen-host interaction. In summary, we developed a standardized genetic transformation platform for *F. oxysporum* featuring three key innovations (**Figure 7**): (1) RSM-optimized enzymatic digestion parameters, (2) selection of phenotypically stable reporter strains, and (3) the development of a platform for functional genome research applicable to *F. oxysporum*. In conclusion, this system could overcome the limitations of conventional histopathological methods and provides a robust framework for studying the pathogenic mechanisms of *Fusarium* species by enabling dynamic tracking of expansion of fungi.

### 4.2 Infection dynamics of *F. oxysporum* in *C. pilosula*: insights from GFP-tagged pathogen tracking

The infection process of *F. oxysporum* involves several key stages: conidial germination, hyphal attachment, epidermal penetration, colonization, and systemic spread (Srivastava et al., 2024; Dai et al., 2016). Successful host recognition and directional growth are critical for pathogenicity (Wu et al., 2022; Amezrou et al., 2024). In this study, GFP-tagged *F. oxysporum* FO-GFP-7 was inoculated onto *C. pilosula* to visualize infection dynamics. At 2 dpi, conidia aggregated at the root epidermis and rhizome, with limited germination into infection hyphae, mirroring early colonization patterns observed in banana roots (Dong et al., 2019). Four days after inoculation, dense hyphal networks were detected in phloem tissues, likely driven by localized nutrient availability, consistent with studies on *Astragalus* root rot (Zhang, 2013). Vascular bundle invasion and tissue maceration at 6 dpi aligned with reports of *F. oxysporum* infections in tomato and *Arabidopsis* (Lagopodi et al., 2002; Guo et al., 2015).

Inoculation via rhizome puncture resulted in a higher incidence of disease compared to unwounded roots, indicating a preference for infection at mechanically damaged crown tissues. This finding aligns with reports by Herman et al. (2008), which documented that *F. oxysporum* targets root hairs in melon, thereby underscoring tissue-specific susceptibility. During bud development, the physiological activity of the rhizome may release chemo-attractants, akin to root exudate-mediated spore recruitment observed in other systems (Bowers et al., 1996). However, conflicting evidence has indicated non-specific epidermal entry in certain hosts (Lebeau et al., 2011; Eynck et al., 2007), highlighting the context-dependent nature of infection strategies.

The results of our observations revealed that while disease incidences were severe, symptom development remained limited in controlled root-inoculated pot trials. This discrepancy likely reflects the absence of natural elements, such as soil microfauna-induced wounds and co-infection stressors that are present under field conditions (Wagner et al., 2021; Dung et al., 2014). Our data confirmed that artificial wounds on rhizomes facilitated pathogen entry and symptom expression, supporting the notion that physical damage accelerates *Fusarium* infections (Savatin et al., 2014). These findings advocate for protective measures to minimize rhizome injuries during cultivation, representing a practical strategy to mitigate root rot in *C. pilosula*.

## 5 Conclusions

This study established an optimized protoplast preparation and genetic transformation system for *F. oxysporum* FO-1, enabling real-time tracking of fungal colonization dynamics in *C. pilosula*. Single-factor experiments combined with response surface methodology identified the optimal enzymatic condition (180 rpm, 4 h, 28°C), which enhanced protoplast yield by 30-fold, achieving 1.44×10^8^ CFU/mL. Eleven GFP-tagged transformants were generated via PEG-mediated transformation, with FO-GFP-7 retaining wild-type morphology, growth rate, and pathogenicity, thereby ensuring biological relevance for infection studies. Fluorescence tracking revealed tissue-specific colonization patterns: conidia aggregated at the rhizome at 2 dpi, followed by phloem infiltration at 4 dpi and vascular invasion at 6 dpi. Notably, wound inoculation at the rhizome accelerated infection progression, mirroring field disease patterns linked to injuries induced by soil microfauna. These findings highlight the rhizome as a critical vulnerability site and underscore the importance of minimizing mechanical damage during cultivation to mitigate root rot. The developed platform provides a robust tool for dissecting *F. oxysporum* pathogenicity and informs targeted strategies for disease management in *C. pilosula* production systems.

## Data Availability

The original contributions presented in the study are included in the article/**Supplementary Material**. Further inquiries can be directed to the corresponding author/s.
