# Supplementary material for: Tracking the infection dynamics of Fusarium oxysporum in Codonopsis pilosula based on GFP labelling
Source: Front Plant Sci. 2025 Aug 15;16:1586118. doi: 10.3389/fpls.2025.1586118 (PMC12394472; doi:10.3389/fpls.2025.1586118)
Supplement: Supplementary file 1 [file DataSheet1.docx]

Supplementary Material

## Supplementary Figures


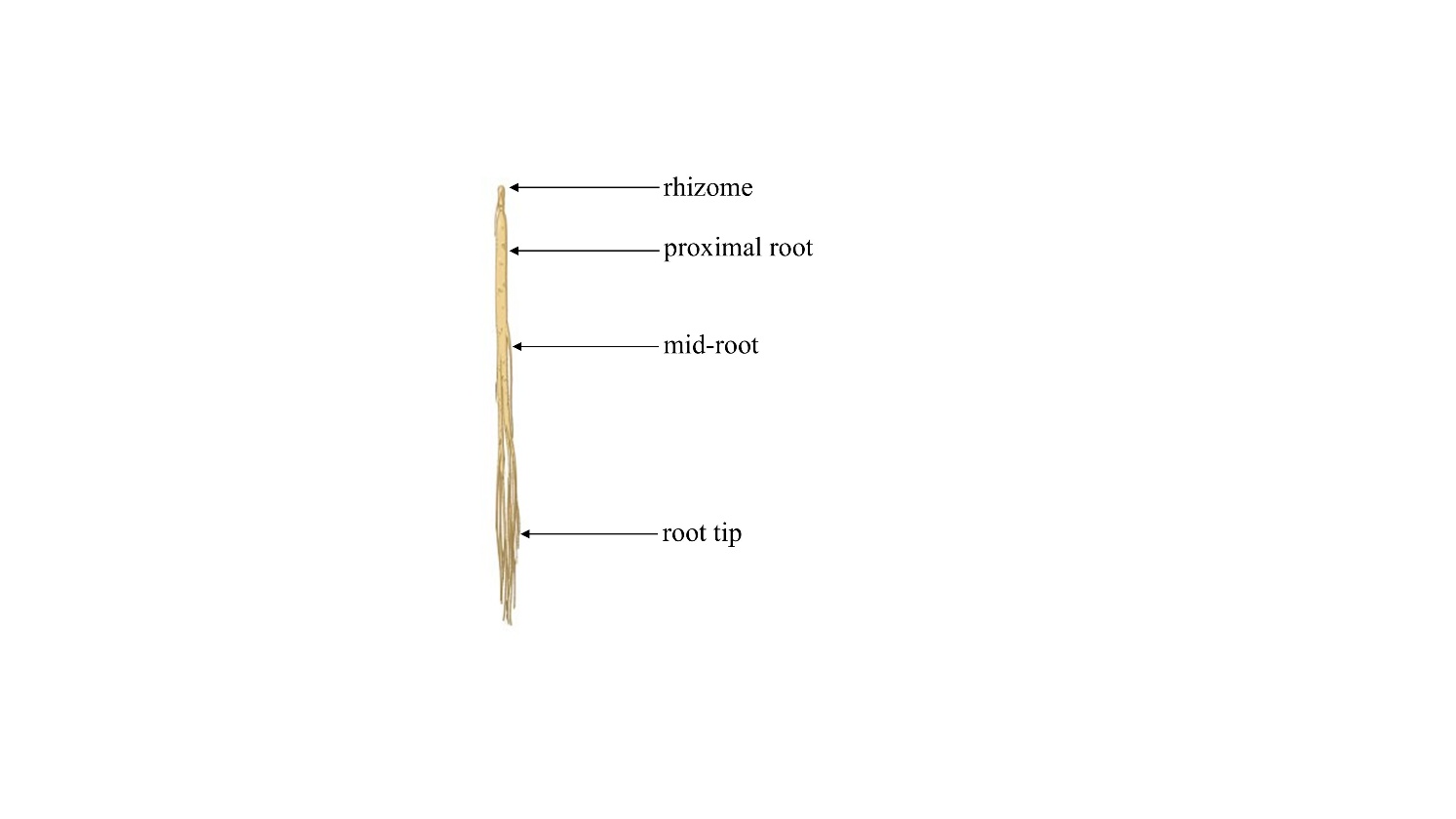


**Supplementary Figure 1.** The locus map of *Codonopsis pilosula* infected by *Fusarium oxysporum*.


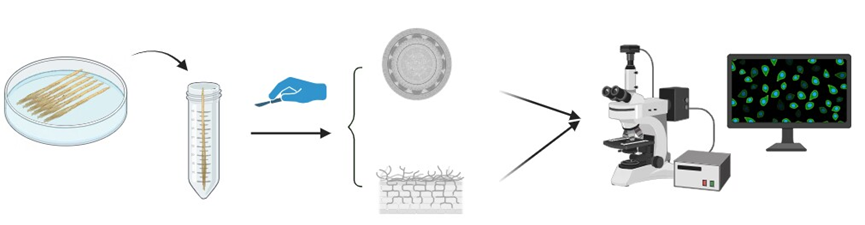


**Supplementary Figure 2.** Flow chart of *Fusarium oxysporum* infecting *Codonopsis pilosula*.

## Supplementary Table

**Table S1.** Response surface test design factors and levels

| **Factor** | **Code** | **Level** | | |
| --- | --- | --- | --- | --- |
|  |  | **-1** | **0** | **1** |
| Enzymolysis time (h) | A | 3 | 4.5 | 6 |
| Enzymolysis temperature (°C) | B | 24 | 28 | 32 |
| Enzymolysis speed (r/min) | C | 140 | 170 | 200 |

**Table S2.** Box-Behnken Design experimental design

| **Test No.** | **A: Enzymolysis time (h)** | **B: Enzymolysis temperature (°C)** | **C: Enzymolysis speed (r/min)** | **Rrotoplast yield**  **(1×10^7^ CFU/mL)** |
| --- | --- | --- | --- | --- |
| 1 | -1 | -1 | 0 | - |
| 2 | -1 | 0 | 1 | - |
| 3 | 0 | -1 | 1 | - |
| 4 | 0 | 1 | -1 | - |
| 5 | 0 | 0 | 0 | - |
| 6 | 0 | 0 | 0 | - |
| 7 | 1 | -1 | 0 | - |
| 8 | 0 | 0 | 0 | - |
| 9 | 0 | 1 | 1 | - |
| 10 | 1 | 0 | 1 | - |
| 11 | 0 | 0 | 0 | - |
| 12 | 0 | -1 | -1 | - |
| 13 | -1 | 0 | -1 | - |
| 14 | 1 | 0 | -1 | - |
| 15 | -1 | 1 | 0 | - |
| 16 | 1 | 1 | 0 | - |
| 17 | 0 | 0 | 0 | - |
